# Rethinking spontaneous giving: Extreme time pressure and ego-depletion favor self-regarding reactions

**DOI:** 10.1038/srep27219

**Published:** 2016-06-02

**Authors:** Valerio Capraro, Giorgia Cococcioni

**Affiliations:** 1 Center for Mathematics and Computer Science (CWI), Amsterdam, The Netherlands; 2 Department of Political Sciences, LUISS University, Rome, Italy

## Abstract

Previous experimental studies suggest that cooperation in one-shot anonymous interactions is, on average, spontaneous, rather than calculative. To explain this finding, it has been proposed that people internalize cooperative heuristics in their everyday life and bring them as intuitive strategies in new and atypical situations. Yet, these studies have important limitations, as they promote intuitive responses using *weak* time pressure or conceptual priming of intuition. Since these manipulations do not deplete participants’ ability to reason completely, it remains unclear whether cooperative heuristics are really automatic or they emerge after a small, but positive, amount of deliberation. Consistent with the latter hypothesis, we report two experiments demonstrating that spontaneous reactions in one-shot anonymous interactions tend to be egoistic. In doing so, our findings shed further light on the cognitive underpinnings of cooperation, as they suggest that cooperation in one-shot interactions is not automatic, but appears only at later stages of reasoning.

The conflict between cooperation and self-interest is one of the most important conflicts in human decision-making. Self-interest is individually optimal, but it may lead to war and destruction; cooperation requires individuals to incur a cost to benefit unrelated others, but it leads to peaceful, healthy, and ultimately more successful societies^1^,^2^,^3^,^4^,^5^,^6^,^7^,^8^.

Formally, cooperation means paying a cost to give a greater benefit to another person^9^. Since bearing a cost to benefit someone else is not individually optimal, additional mechanisms are needed for the evolution of cooperation among self-interested subjects. Five such mechanisms have been proposed^9^: (i) if interactions are repeated, cooperation may be optimal in the long run, because the cost of one’s cooperation today may be outweighed by the benefit of partner’s reciprocal cooperation tomorrow; (ii) if interactions are structured, rather than randomly mixed, cooperators may form clusters and protect themselves from the invasion of defectors; (iii) if interactions are between genetically related individuals, cooperation is optimal if the coefficient of relatedness between individuals exceeds the cost-to-benefit ratio, because people care about related others proportionally to their coefficient of relatedness; (iv) if interactions are between groups, rather than between individuals, then cooperation can evolve within a group, because cooperative groups can generate a larger payoff and outcompete non-cooperative ones; (v) if interactions are repeated and can be observed by third-parties, then it may be optimal to cooperate today to gain a good reputation and earn the benefit of someone’s else reciprocal cooperation tomorrow.

Yet, many people cooperate also in one-shot anonymous interactions^10^,^11^,^12^,^13^,^14^ and even in large groups^15^,^16^, despite the fact that none of the five rules of cooperation is at play: by definition, one-shot anonymous interactions are not repeated, are not structured, are not with genetically related partners, are not between groups, and cannot be observed by others. Why are some people willing to pay a cost to help a stranger when no future direct or indirect reward is at stake?

Over recent years, there has been increasing interest in studying cooperation in one-shot anonymous interactions from a dual-process perspective. Dual-process theories^17^,^18^,^19^,^20^,^21^ posit that human decisions result from the interaction between two cognitive systems, one that is quick, automatic, and intuitive, named System 1, and one that is slow, controlled, and deliberative, named System 2. Adopting this lens raises the following question: is cooperation in one-shot anonymous interactions automatic, or does it require deliberation^22^?

Previous research suggests that cooperation in one-shot anonymous interactions is spontaneous and subjects become greed only after deliberation^22^,^23^,^24^,^25^,^26^,^27^ (and see ref. 28 for a meta-analysis). To explain these results, Rand and colleagues^23^ have introduced the Social Heuristics Hypothesis (SHH), which maintains that people internalize strategies that are successful in their everyday life and bring them as default strategies in new and atypical situations. Then, after deliberation, they may override these heuristics and shift their behavior towards the one that is optimal in the given situation.

The fact that this theoretical account predicts that promoting intuition versus deliberation has indeed a positive effect on cooperation in one-shot anonymous interactions, can be easily shown as follows. Since most everyday interactions are with family members, friends, and co-workers, and they are thus networked and repeated, cooperation may evolve in daily life thanks to the aforementioned five rules of cooperation. Thus, on the one hand, the SHH predicts that people may internalize cooperative heuristics in their everyday life, and bring them as intuitive strategies in the new and atypical situation of a one-shot anonymous laboratory experiments. But, on the other hand, the SHH also predicts that subjects, after deliberation, override their heuristics and understand that cooperating in one-shot anonymous interactions is not optimal, because no direct or indirect rewards are at play. Thus the SHH predicts that deliberation makes subjects switch from cooperation to defection. In this theoretical framework, cooperation in one-shot anonymous interactions is thus explained as an overgeneralization of a behavior learned in everyday interactions and transferred to one-shot interactions, because “the intuitions and norms that guide these decisions were shaped outside the laboratory by mechanisms for the evolution of cooperation”^7^.

There are, however, two reasons to think that this theorization may be incomplete and that, in particular, spontaneous choices in one-shot anonymous interactions may not be as cooperative as it predicts.

One is that the SHH assumes that subjects, when facing new and atypical situations, can apply, automatically and without any effort, cooperative heuristics that have been shaped *outside* the situation they are currently facing (i.e., in everyday interactions). The assumption that heuristics learned in setting X can be applied automatically and without any effort to a different setting Y is questionable, because subjects, before applying heuristics shaped in setting X to the new setting Y, must first recognize the similarity between X and Y. Recognizing this similarity may not come for free and may require a small but positive amount of cognitive effort. This view is in fact consistent with Kohlberg’s rationalist approach^29^,^30^,^31^,^32^, which assumes that the application of internalized rules and norms happens only at the second, *conventional*, level of reasoning, which requires a non-zero amount of cognitive effort needed to overcome the primal and egoistic impulse which, according to Kohlberg, characterizes the first, *pre-conventional*, level of reasoning.

The second one is that cooperation in one-shot interactions may also emerge from the application of abstract ethical principles, such as the Golden Rule-treat others as you would like others treat you-which encapsulates the essence of cooperative behavior and is “found in some form in almost every ethical tradition”^33^. According to Kohlberg’s rationalism^29^,^30^,^31^,^32^, abstract ethical principles are applied only at the third, *post-conventional*, level of reasoning, requiring a high amount of cognitive resources. In line with this view, a recent experiment has shown that time pressure decreases cooperation among subjects with high cognitive abilities^34^.

Taking these observations into account, one should expect that automatic reactions in one-shot anonymous interactions be self-regarding, rather than cooperative. This should be true especially for *naïve* subjects, those with no previous experience in experimental games involving cooperation with an anonymous stranger. There are in fact two (related) reasons for predicting a moderating role of experience. On the one side, previous research has shown that level of experience decreases treatment effects, essentially because, as subjects become increasingly experienced with an experimental paradigm, it becomes more difficult to manipulate their behavior^23^,^35^,^36^. The second one is that the crucial point of our argument is that cooperative heuristics are not automatic because they are shaped *outside* the situation which a subject is currently facing, and thus the subject needs to spend a non-zero cognitive effort to recognize the similarity between the situation she or he is currently facing and the situation in which her or his heuristics have been shaped. But, if a subject is already experienced with our experimental paradigm, she or he may have developed *internal* heuristics, which can be accessed at zero cognitive effort, because they are shaped within the same context. Summarizing, in this work we wish to test the following hypothesis.

*Hypothesis.* Automatic responses in one-shot anonymous cooperation games are self-interested, particularly among subjects with no previous experience in experimental games involving cooperation with an anonymous stranger.

It is important to note that previous studies can neither be used to reject nor support this hypothesis. Indeed, to the best of our knowledge, previous research, investigating the role of cognitive manipulation on cooperation in one-shot anonymous interactions, has used either *light* time pressure (forcing subjects to make a decision within 10 seconds) or conceptual priming of intuition to favor intuitive choices over deliberative ones. These studies confirm that promoting intuition versus reflection increases cooperative choices in one-shot anonymous interactions, but only among naïve subjects with high levels of interpersonal trust in the setting where they live^22^,^23^,^35^,^37^. While these results are in line with (and, in fact, inspired) the Social Heuristics Hypothesis, they do not contradict our hypothesis, since light time pressure and conceptual priming of intuition are not powerful enough to detect automatic and effortless responses. Specifically, 10 seconds is a relatively long decision time, during which subjects are likely to be able to perform a reasoning of non-zero cognitive complexity; and, similarly, conceptual priming of intuition does not deplete cognitive complexity, but it only makes subjects more likely to rely on their intuitions.

Thus, to shed light on our hypothesis, we carried out a series of experiments, in which all subjects played a one-shot Prisoner’s Dilemma (PD) game under different conditions. In our PDs, subjects are given a certain amount of money and asked to decide how much, if any, to give to another anonymous participant. Every unit transferred would be multiplied by 2 and earned by the other participant. Thus, both participants would be better off if they both transfer money to each other (cooperate), than if they both keep the money (defect). However, each participant is better off by keeping the money, no matter what the other is doing. For this reason, the proportion of the endowment transferred to the other participant is usually taken as an individual measure of cooperative attitudes.

## Results

### Study 1: Extreme time pressure decreases cooperation in one-shot anonymous interactions

Our first experiment aims at showing that forcing subjects to make extremely quick choices decreases cooperation in one-shot anonymous Prisoner’s Dilemma experiments.

Classically, it has been assumed that time pressure impairs participants’ ability to reason about the details of the situation and thus increases the likelihood that they use readily available strategies^17^,^18^,^19^,^20^,^21^,^23^,^38^,^39^. However, previous studies^22^,^23^,^24^, exploring the effect of time pressure on cooperation in one-shot interactions, have induced quick decisions by putting a 10 seconds time pressure on the decision screen (separated from the instruction screen). This procedure has the main limitation that subjects can stay on the instruction screen as long as they want, and use this window of time to reason about the decision problem, before moving on to the decision screen and actually make a decision. Additionally, 10 seconds is a relatively long decision time, during which subjects are likely to be able to perform a reasoning of non-zero cognitive complexity. Thus it is likely that the choices analyzed in these studies are not as spontaneous and automatic as it is argued therein.

To overcome these problems, in our experiment, time constraints were added directly on the instruction screen (which coincided with the decision screen). Subjects in the *time pressure* condition were asked to make a decision within 10 seconds; those in the *time delay* condition were asked to think for at least 30 seconds before making their choice. A visible timer recorded the time spent on this screen. Right after making their decision, participants were asked four comprehension questions to make sure they understood the social dilemma structure of the game. Participants failing any comprehension question were automatically eliminated from the survey. Those who passed the comprehension questions entered the demographic questionnaire, where, along with the usual questions (sex, age, level of education), they were asked to self-report the extent to which they have already participated in experiments involving exchanging money with an anonymous stranger. We collected responses using a 5-point Likert-scale from “never” to “very often”. This is the same measure of experience used in previously published studies^13^,^23^,^35^,^37^. As in these studies, we say that a participant is naïve if their answer was “never”. We refer the reader to the Methods for more details about the design and to the Supplementary Information for full experimental instructions.

A total of 837 subjects, living in the US and recruited through Amazon Mechanical Turk^40^,^41^,^42^,^43^, passed the comprehension questions. Among these, 355 acted under time pressure and 482 acted under time delay. This differential attrition was caused by the fact that participants under time pressure were more likely than those under time delay to fail the comprehension questions. Such a differential attrition is potentially problematic, because it may lead to selection problems. We will address this point at the end of this subsection.

**Figure 1:**
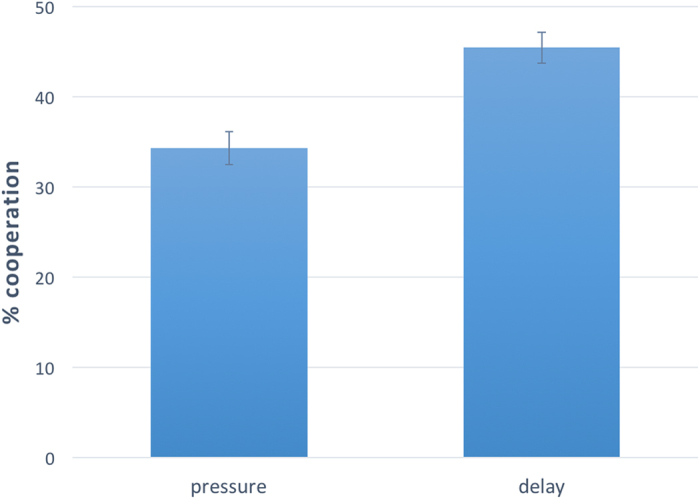
Extreme time pressure favors selfish behavior on the whole sample. Results of Study 1 (N = 837). Subjects forced to make an extremely quick choice are significantly more selfish than those forced to stop and think about their choice. Linear regression predicting “cooperation” as a function of a dummy variable, named “pressure”, taking value 1 if the subject participated in the time pressure condition, and 0 if the subject participated in the time delay condition, confirms the presence of a significant and negative effect of “pressure” on “cooperation” with (F(4,831) = 8.254, coeff = −0.110, p < 0.0001, r^2^ = 0.039) and without (F(1,835) = 19.502, coeff = −0.111, p < 0.0001, r^2^ = 0.023) control on sex, age, and level of education. Error bars represent the standard error of the mean.

Coming to our research question, we now explore whether average cooperation among subjects acting under time pressure was different than average cooperation among subjects acting under time delay. We start by analyzing experienced and naïve subjects together. To avoid selection bias, we include in our analysis also subjects who did not obey the time constraints^37^,^44^. Figure 1 summarizes the effect of reaction time manipulation on cooperative behavior and provides visual evidence that subjects forced to make quick decisions act more selfishly than those forced to stop and think about their decision. Linear regression predicting average cooperation as a function of a dummy variable, named “pressure”, taking value 1 if the subject participated in the time pressure condition, and 0 if the subject participated in the time delay condition, confirms the presence of a significant and negative effect of “pressure” on “cooperation” (F(1,835) = 19.502, coeff = −0.111, p < .0001, r^2^ = 0.023), which is robust after controlling for sex, age, and level of education (F(4,831) = 8.254, coeff = −0.110, p < 0.0001, r^2^ = 0.039).

**Figure 2:**
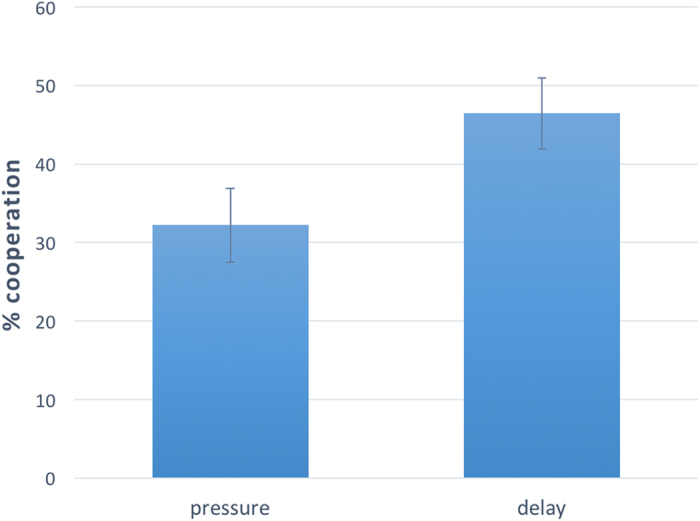
Extreme time pressure favors selfish behavior on naïve subjects. Results of Study 1, restricted to naïve subjects (N = 119). Subjects forced to make an extremely quick choice are significantly more selfish than those forced to stop and think about their choice. Linear regression predicting average cooperation as a function of a dummy variable, named “pressure”, taking value 1 if the subject participated in the time pressure condition, and 0 if the subject participated in the time delay condition, confirms the presence of a significant and negative effect of “pressure” on “cooperation” with (F(4,115) = 1.408, coeff = −0.151, p = 0.028, r^2^ = 0.047) and without (F(1,118) = 4.570, coeff = −0.142, p = 0.035, r^2^ = 0.037) control on sex, age, and level of education. Error bars represent the standard error of the mean.

Next we explore the moderating role of experience. Figure 2 shows that, when we restrict the analysis to naïve subjects only, the effect of reaction time manipulation on cooperation remains in the same direction, and even increases in size, passing from about 10% (whole sample) to about 15% (naïve subjects only). Linear regression confirms that the effect of “pressure” on “cooperation” is still negative and significant (with control: F(4,115) = 1.408, coeff = −0.151, p = 0.028, r^2^ = 0.047; without control: F(1,118) = 4.570, coeff = −0.142, p = 0.035, r^2^ = 0.037). However, although the negative effect of time pressure on cooperation, when passing from the whole sample to naïve subjects only, increases in size, linear regression predicting “cooperation” as a function of “pressure”, “naivety”, and their interaction reveals that the interaction term is not significant (p = 0.616). This suggests that reaction time manipulation had the same negative effect on all participants, regardless of their level of experience.

Next we address the problem whether our results may be driven by differential attrition. To this end, we observe that there are 127 more subjects in the time delay condition than in the time pressure condition. How should these 127 “missing” subjects behave, in order to be able to make the above correlation insignificant? To answer this question, we complete the sample of subjects acting under time pressure by adding 127 subjects, each of whom transferring a proportion q of their endowment to the other participant, and we estimate the smallest q such that linear regression on the resulting, completed, sample would reveal non-significant results. In doing so, we find approximatively q = 0.6. More precisely, with exactly q = 0.6, the p-value resulting from the linear regression would be p = 0.0502. In other words, in order to make the above correlation non-significant, one has to assume that subjects who failed the comprehension questions in the time pressure condition would have cooperated with probability at least 0.6, had they passed the comprehension questions. Although this is certainly possible, it is unlikely since average cooperation in our study is well below 50% and thus it is not clear why would-be cooperators should be more likely than would-be defectors to fail the comprehension questions in the time pressure condition.

In sum, Study 1 provides evidence that extreme time pressure decreases cooperative behavior in one-shot anonymous prisoner’s dilemmas, independently of participants’ level of experience in economic games involving exchanging money with an anonymous stranger.

### Study 2: Ego-depletion decreases cooperation in one-shot anonymous interaction, but only among naïve subjects

Study 1 used time pressure to favor automatic reactions over deliberative ones, and provided evidence for the hypothesis that automatic responses in one-shot anonymous prisoner’s dilemmas are self-regarding. One potential limitation of Study 1 is the very use of time pressure to induce intuitive responses. If, on the one hand, it has classically been assumed that time pressure indeed promotes intuitive thinking^17^,^18^,^19^,^20^,^21^,^23^,^38^,^39^, on the other hand, recent studies have pointed out that fast reaction times may not be a good proxy for intuitive thinking, because response speed interacts with decision conflict^45^, strength of preferences^46^, and subjects’ probability to make a mistake in implementing their strategy^47^. Although these studies have, so far, challenged the use of reaction times and not the use of time manipulations, they caution against the use of time pressure to induce intuitive thinking. To address this issue, Study 2 tests the same hypothesis as Study 1, but using a different cognitive manipulation.

Specifically, Study 2 uses ego-depletion to disentangle automatic choices from calculative ones. Ego-depletion is a cognitive manipulation based on the self-regulatory strength model, which posits that all acts of self-control draw on a common resource. Although cognitive scientists are still debating on whether this resource is finite^48^ or infinite^49^, theoretical and experimental research converge on the fact that performing a difficult task, requiring self-control, results in a poor performance in a subsequent unrelated self-control task^48^,^49^,^50^,^51^,^52^,^53^,^54^: if not sufficiently stimulated, depleted subjects tend to mentally rest in the subsequent task and thus they are “more apt to act on impulse”^48^.

A few recent studies have investigated the role of ego-depletion on numerous games involving cooperative behavior. However, these studies have been conducted either on iterated games^55^,^56^, or on games where cooperating with one player implies competing with a third player^57^. To the best of our knowledge, no study has been conducted on one-shot anonymous Prisoner’s Dilemma games. As mentioned in the Introduction, using one-shot games, rather than repeated ones, is fundamental for testing our hypothesis, since repeated interactions permit the evolution of *internal* heuristics, which can be accessed with zero cognitive effort.

To fill this gap, we conducted an experiment in which (brand new) subjects were randomly divided between two conditions. In the *depletion* (resp. *no-depletion*) condition, they had to complete a cognitively demanding (resp. easy) task, before playing a one-shot anonymous Prisoner’s Dilemma. The remaining part of the study was identical to Study 1. We refer to the Methods section for more details about the design and to the Supplementary Information for full experimental instructions.

As ego-depletion tasks we employed three different tasks: the Stroop task^58^, the *e*-hunting task^59^, and the give-the-wrong-answer task (see Methods). Our initial motivation for changing depletion task was that our first experiment, using the Stroop task, gave rise to a differential attrition similar to Study 1: depleted subjects were more likely than non-depleted subjects to leave the experiment during the task or fail the comprehension questions (drop-out rates: 58% in the depletion condition, 29% in the no-depletion condition). The other two tasks did not give rise to differential attrition: in the *e*-hunting task, the drop-out rate among depleted subjects was 50%, while the drop-out rate among non-depleted participants was 47% (rank sum, p = 0.834). In the give-the-wrong-answer task, the drop-out rate among depleted subjects was 51%, while the drop-out rate among non-depleted participants was 46% (rank sum, p = 0.215). Moreover, aggregating the results of the three experiments and predicting cooperation as a function of “experiment” (1 = Stroop task, 2 = *e*-hunting task, 3 = give-the-wrong-answer task) and “depletion” (0 = no depletion condition, 1 = depletion condition) and their interaction, we find that the interaction term is not significant (p = 0.394), suggesting that the depletion task had a similar effect on the three experiments, in spite of the fact that, in the first experiment, it gave rise to differential attrition.

**Figure 3:**
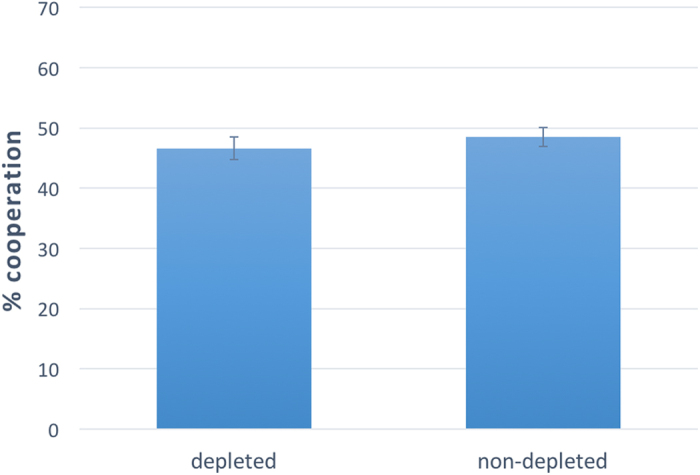
Ego-depletion has no effect on cooperation on the whole sample. Results of Study 2 (N = 1,157). Ego-depletion has no effect on cooperation on the whole sample (naïve and experienced subjects together). Linear regression predicting “cooperation” as a function of a dummy variable, named “depletion”, which takes value 1 if a subject participated in the depletion condition, and 0 if the subject participated in the no-depletion condition, confirms that there is no significant effect (without control on sex, age, and education: F(1,1155) = 0.589, coeff = −0.020, p = 0.443, r^2^ = 0.000; with control: F(4,1152) = 0.736, coeff = −0.019, p = 0.448, r^2^ = 0.002). Error bars represent the standard error of the mean.

Now we pass to the analysis of the effect of ego-depletion on cooperative behavior. Since the interaction between “experiment” and “depletion” was not significant, we can aggregate the data of the three experiments. Figure 3 provides visual evidence that depleting participants’ self-control resources has no effect on cooperation, when one does not take into account participants’ level of experience. This is confirmed by linear regression predicting “cooperation” as a function of “depletion” (without control on sex, age, and education: F(1,1155) = 0.589, coeff = −0.020, p = 0.443, r^2^ = 0.000; with control: F(4,1152) = 0.736, coeff = −0.019, p = 0.448, r^2^ = 0.002).

**Figure 4:**
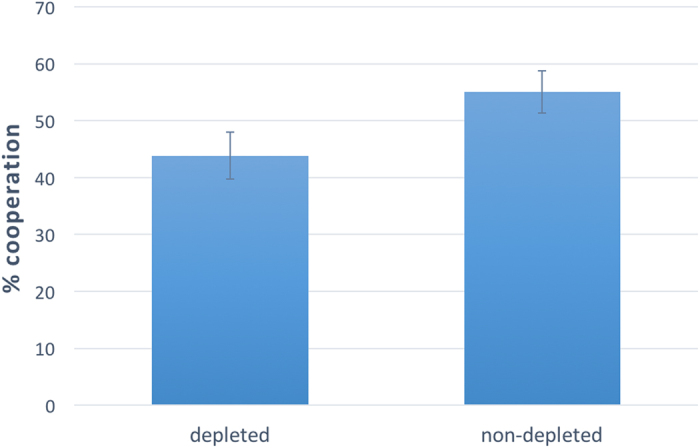
Ego-depletion decreases cooperative behavior among naïve subjects. Results of Study 2, restricted to naïve subjects (N = 231). Depleted participants are significantly more selfish than non-depleted ones. Linear regression predicting “cooperation” as a function of a dummy variable, named “depletion”, which takes value 1 if a subject participated in the depletion condition, and 0 if the subject participated in the no-depletion condition, confirms that there is a significant and negative effect of “depletion” on “cooperation” with (F(4,227) = 1.858, coeff = −0.114, p = 0.041, r^2^ = 0.032) and without (F(1,230 = 4.088, coeff = −0.112, p = 0.044, r^2^ = 0.017) control on sex, age, and education. Error bars represent the standard error of the mean.

Next, we explore the moderating role of naivety. Figure 4 reports the effect of ego-depletion on naïve subjects only and provides visual support for the existence of a negative effect. To show this, we first conduct linear regression predicting “cooperation” as a function of “naivety”, “depletion”, “experiment”, and all their two-way interactions. This analysis shows a marginally significant effect of the interaction “naivety x depletion” (without control: coeff = −0.109, p = 0.077; with control: coeff = −0.111, p = 0.071), suggesting that the effect of ego-depletion on cooperation among naïve subjects was different from its effect on experienced subjects. To further explore this, we restrict the analysis to naïve subjects only and we conduct linear regression predicting “cooperation” as a function of “depletion”. The analysis indeed uncovers a significant and negative effect of ego-depletion on cooperative behavior (without control: F(1,230 = 4.088, coeff = −0.112, p = 0.044, r^2^ = 0.017; with control: F(4,227) = 1.858, coeff = −0.114, p = 0.041, r^2^ = 0.032. Effect sizes: Stroop task = 8.8%; *e*-hunting task = 12.7%; give-the-wrong-answer task = 12.3%)

In sum, Study 2 shows that ego-depletion interacts with participants’ level of experience, such that ego-depletion impairs cooperative behavior in one-shot anonymous prisoner’s dilemma interactions among naïve subjects, but not among experienced ones.

## Discussion

We have shown that automatic and effortless reactions in one-shot anonymous Prisoner’s Dilemma games tend to be egoistic, particularly among subjects with no previous experience in experiments involving exchanging money with anonymous strangers.

Specifically, Study 1 showed that extreme time pressure decreases cooperative behavior in one-shot anonymous Prisoner’s Dilemma games. The effect was statistically significant both at the level of the whole sample and when restricting the analysis to naïve subjects only. Although our design led to differential attrition, according to which subjects under time pressure were more likely to fail the comprehension questions than those under time delay, we showed that the size of the negative effect of time pressure on cooperation is so big that it is unlikely that it is driven by differential attrition. Study 2 showed that ego-depletion decreases cooperative behavior in one-shot Prisoner’s Dilemma games, but only among naïve subjects.

Both time pressure and ego-depletion are thought to favor automatic and effortless reactions in experimental subjects. Time pressure impairs participants’ ability to reason about the details of the situation and thus it increases the likelihood that subjects use readily available strategies^17^,^18^,^19^,^20^,^21^,^23^,^38^,^39^. Regarding ego-depletion, theoretical and experimental research converge on the fact that performing a difficult task, requiring self-control (i.e., being depleted of one’s own ego), results in a poor performance in a subsequent unrelated self-control task, in which, if not sufficiently rewarded, participants tend to rest and choose readily available strategies^48^,^49^,^50^,^51^,^52^,^53^,^54^. Thus, taken together, our two studies provide evidence that automatic reactions in one-shot anonymous Prisoner’s Dilemma games tend to be egoistic, particularly among naïve subjects.

In this light, our results are in line with recent experimental research showing that time pressure decreases cooperative behavior among subjects with high cognitive abilities^34^, that self-control benefits cooperation^60^, and that sleep restriction decreases trustworthiness^61^. In terms of theoretical framework, our results are consistent with Kohlberg’s Rationalist Approach^29^,^30^,^31^,^32^, which states that moral decisions result from one of three hierarchical levels of reasoning, requiring increasing levels of cognitive abilities. The lowest *pre-conventional* level is characterized by primal, egoistic reactions; the intermediate *conventional* level involves the application of internalized rules and norms; and the advanced *post-conventional* moral reasoning involves the application of abstract and universal ethical principles. Thus, according to Kohlberg, automatic and effortless responses in one-shot anonymous prisoner’ dilemmas should be egoistic (pre-conventional level); cooperation may emerge at intermediate levels of cognitive effort, thanks to the emergence of cooperative heuristics (conventional level); and increase even more at high levels of cognitive effort, thanks to the application of abstract and universal ethical principles, such as the Golden Rule (post-conventional level). Thus, Kohlberg’s rationalist approach predicts a positive correlation between cognitive complexity and cooperation.

Our data suggest also a moderating role of level of experience: ego-depletion had a negative effect on cooperation only on naïve subjects. One potential explanation for this moderating role of level of experience is that experienced participants are less subject to treatment effects. This is a well-known problem with Mechanical Turk experiments, particularly when the experimental conditions aim at disentangling automatic responses from deliberative ones^23^,^35^,^36^,^37^. Additionally, although Kohlberg’s rationalist approach is silent about the amount of deliberation needed to access internalized rules and norms (belonging to the conventional level), it is possible that it depends on the level of experience, such that experienced subjects need less cognitive effort to reach their heuristics. Thus, in principle, a moderating role of experience is consistent with Kohlberg’s theoretical framework.

With the few exceptions mentioned above^34^,^60^,^61^, the vast majority of previous research has shown that time pressure^22^,^23^,^24^,^27^,^28^ and conceptual priming of intuition^23^,^25^ tend to increase cooperation in one-shot cooperation games, particularly among naïve subjects (see also refs 42 and 62 for null results). To explain these results, it was proposed that subjects internalize cooperative heuristics in their everyday interactions and bring them as intuitive strategies in new and atypical situations (such as lab experiments for naïve subjects). Then, after additional deliberation, subjects may override these heuristics and shift their behavior towards the one that is optimal in the given situation^23^. Numerous predictions of this *Social Heuristics Hypothesis (SHH)* have been successfully shown, including that time constraints have effect only on subjects with high levels of trust in the setting where they live^35^, have no effect on subjects living in a country with low levels of interpersonal trust^37^, and that intuition promotes altruism for women but not for men^63^. Moreover, the SHH has also been supported by a recent game theoretical model of dual-process players, which predicts that evolution never favors strategies that intuitively defects and deliberately cooperate^64^.

Thus our result seems to contradict previous experimental findings. To explain this apparent divergence, we note that previous experiments have investigated the effect of *light* time pressure and conceptual priming of intuition on cooperation. On the other hand, here we have explored the effect of *extreme* time pressure and ego-depletion, both of which deplete subjects’ ability to deliberate to a greater extent than light time pressure and conceptual priming of intuition. Thus, it is possible that previous studies, reporting spontaneous cooperation, uncovered only the “decreasing part” of an inverted-U relationship.

To be more precise, one can speculate that heuristics, learned in typical and familiar situations, interact with behavior in new and atypical situations through a non-linear model which integrates Kohlberg’s rationalist approach and the Social Heuristics Hypothesis as follows: (i) according to Kohlberg’s approach, automatic and effortless responses are self-regarding, but only in new and atypical situations; (ii) in typical and familiar situations, moral norms can evolve through a number of mechanisms (e.g., the five rules for the evolution of cooperation) and, according to the SHH, they can get internalized as heuristics; (iii) when people face a new and atypical situation, they tend to rely on these heuristics, as long as they make, or have the ability to make, the minimum cognitive effort needed to recognize the similarity between the new situation they are facing and the typical situation in which those heuristics have been shaped; (iv) when people face a new and atypical situation in a condition of ego depletion or extreme time pressure, they act according to their automatic egoistic impulse; (v) when the ability to make a cognitive effort is not depleted, people get access to their heuristics and to their moral motivations (according to Kohlberg’s rationalism), and, consequently, they may start cooperating; (vi) after additional deliberation, individuals may override their heuristics (according to the SHH) and their moral motivations, via moral rationalization^65^ or the *sheer force of logic*^66^, and adjust their behavior towards the one that is optimal in the given situation.

This model predicts the existence of an inverted-U relationship between cognitive effort and cooperation which would be consistent with the apparently contradictory findings that, particularly among naïve subjects, extreme time pressure and ego-depletion decreases cooperation (our result and that of ref. 61), while light time pressure and conceptual priming of intuition increases cooperation (previous results). Although compelling, we cannot fully support this model, as our results do not provide direct evidence for an inverted-U effect of cognitive effort on cooperation. However, interestingly, we note that the idea that cognitive complexity may have an inverted-U effect on moral choices, more generally, is not new, as it was recently proposed by Moore and Tenbrunsel^67^. Investigating this and other potential theoretical frameworks for this body of experimental research is certainly an important direction for future work, given the importance that understanding the cognitive underpinning of human cooperation may have on designing institutions to promote cooperative behavior.

In any case, our main result seems to go against the predictions of a theoretical model recently proposed by Bear and Rand^64^. In this model, dual process subjects interact in either one-shot or repeated interactions: intuitive subjects always choose the same strategy, independently of whether the interactions are repeated or one-shot; deliberative subjects pay a cost to deliberate and choose the strategy that is optimal in the given interaction. The authors found that natural selection always favors either intuitive defectors, who never deliberate, or dual-process subjects who intuitively cooperate and sometimes use deliberation to defect in one-shot games. Thus, this model makes the explicit prediction that deliberation can never increase cooperative behavior. This prediction has not been confirmed by our results. However, we note that Bear and Rand’s model implicitly assumes that heuristics are completely automatic and effortless. This assumption is likely not to apply to our case, since the heuristics of naïve subjects are shaped outside the situation they are currently facing. Thus, getting access to their heuristics may not come for free and subjects may need a small but positive amount of cognitive effort. Further studies may help understand whether our results are consistent with a variant of Bear and Rand’s model in which subjects need a small but positive amount of cognitive effort to get access to their heuristics.

## Methods

We conducted two experiments recruiting subjects, residents in the US, using Amazon Mechanical Turk (AMT). AMT studies are easy to program and fast and cheap to realize, as participants are paid only a few cents for completing a survey, which normally takes five minutes or even less. Questions may arise, then, regarding the reliability of data collected using such small stakes. Previous research suggests that data collected using AMT are of no less quality than data collected using standard laboratory experiment^40^,^41^,^42^,^43^. The statistical equivalence between data gathered on AMT and those collected on laboratory experiments includes also games involving pro-social behavior, as the ones considered in this work. For example, it has been shown that average cooperation in a one-shot Prisoner’s Dilemma played online is statistically indistinguishable from average cooperation in the same game played in the physical laboratory with 10 times larger stakes^41^, and that average altruism in a Dictator game played online is essentially the same as the one reported in Engel’s meta-analysis^68^ of 616 Dictator game experiments (26.7% vs 28.3%)^69^.

### Study 1

Participants earned $0.40 for participating. All participants were shown an introductory screen, in which they were told that they would be paired with another anonymous participant and that both participants would be shown the same screens. After this introductory screen, subjects entered the instructions/decision screen in either the *time pressure* or the *time delay* condition. Each subject was given $0.20 and was asked to decide how much, if any, to transfer to the other player. Each cent transferred would be doubled and earned by the other player. Subjects in the time pressure condition were asked to make a decision within 10 seconds; those in the time delay condition were asked to stop and think for at least 30 seconds before making their decision. The time constraints were put directly on the instruction screen and a visible timer recorded the amount of time each participant spent on this screen. After the decision was made, four comprehension questions were asked to make sure that subjects had understood the social dilemma. Subjects failing any comprehension question were automatically excluded from the survey. A self-report inventory completed the survey. A part from asking standard demographic questions (sex, age, and education), here we measured participants’ level of experience by asking them to what extent they had already participated in surveys involving exchanging money with an anonymous stranger. We collected answers using a 5-point Likert scale from 1 (never) to 5 (very often). We refer the reader to the Supplementary Information for full experimental instructions.

### Study 2

Our second study was made of three single experiments, differing on the task used to deplete participants’ self-control resources.

#### Stroop task

Participants earned $0.50 for participating and were randomly assigned to either the *no-depletion* condition or the *depletion* condition. In the *no-depletion* condition, they had to complete a very easy Stroop task consisting of 20 items. Each item consisted of a color word (e.g., blue), written in the same color, and subjects were asked to type the color of the word. In the *depletion* condition, each item consisted of a color word (e.g., blue), but written in a different color (e.g., red), and subjects were asked to type the color of the word. Participants failing to report the right color were not allowed to go to the next screen. After the Stroop task, participants were paired together to play a one-shot Prisoner’s Dilemma, as in Study 1. The rest of the experiment was identical to Study 1. We refer the reader to the Supplementary Information for full experimental instructions.

#### e-hunting task

The procedure was almost identical to that of the experiment using the Stroop task. The only difference is that we used the *e*-hunting task, instead of the Stroop task, to deplete participants’ ability to put cognitive effort into the subsequent Prisoner’s Dilemma. The *e*-hunting task is a two-stage task: the first stage is identical for both the depletion and the no-depletion conditions and consists of a neutral piece of text (in our case, randomly chosen sentences from a textbook for an advanced course in pure mathematics), in which subjects have to find all instances of the letter *e*. After this first stage of the task, finding *e*s in a piece of text becomes an automatic response. In the second part of the task, all participants are given another piece of text, but the task depends on the condition. In the *no-depletion* condition, participants have to find, again, the letter *e*. In the *depletion* condition, they have to find all instances of the letter *e*, that are not one letter away from another vowel (so, for example, the letter *e* in the word *vowel* does not count, since it is one letter away from the vowel *o*). Thus, depleted participants have to override the automatic response learned in the first part of the task to find the right answer. In all tasks, participants making an error larger than 5% of the correct number of *e*s to be found were not allowed to go to the next screen. We refer the reader to the Supplementary Information for full experimental instructions.

#### Give-the-wrong-answer task

The design was very similar to the previous ones. The only differences were in the task used to deplete subjects’ self-control resources and the addition of a self-report question, right after the ego depletion manipulation, in which we asked participants the extent to which they felt tired. Moreover, since the depletion task was longer than the ones used in the previous experiment, we increased the participation fee up to $1. As ego depletion task we used a 30-item give-the-wrong-answer task. In this task, each item consists of a very easy question (e.g., What is the name of Angelina Jolie’s husband?) with two possible answers (e.g., Brad Pitt or Sean Connery). Subjects in the *no-depletion* condition were asked to tick the right answer; participant in the *depletion* condition were asked to tick the wrong answer. We refer the reader to the Supplementary Information for full experimental instructions.

According to the Dutch legislation, this is a non-WMO study, that is (i) it does not involve medical research and (ii) participants are not asked to follow rules of behavior. See http://www.ccmo.nl/attachments/files/wmo-engelse-vertaling-29-7-2013-afkomstig-van-vws.pdf, §1, Article 1b, for an English translation of the Medical Research Act. Thus (see http://www.ccmo.nl/en/non-wmo- research) the only legislations which apply are the Agreement on Medical Treatment Act, from the Dutch Civil Code (Book 7, title 7, §5), and the Personal Data Protection Act (a link to which can be found in the previous webpage). The current study conforms to both. Informed consent was obtained by all subjects prior to participating.

## Additional Information

**How to cite this article**: Capraro, V. and Cococcioni, G. Rethinking spontaneous giving: Extreme time pressure and ego-depletion favor self-regarding reactions. *Sci. Rep.* **6**, 27219; doi: 10.1038/srep27219 (2016).

## Supplementary Material

Supplementary Information
