# Supplementary material for: Rethinking spontaneous giving: Extreme time pressure and ego-depletion favor self-regarding reactions
Source: Sci Rep. 2016 Jun 2;6:27219. doi: 10.1038/srep27219 (PMC4890119; doi:10.1038/srep27219)
Supplement: Supplementary Information [file srep27219-s1.pdf]

Supplementary Information

Rethinking spontaneous giving: Extreme time pressure and ego-depletion favor self-regarding reactions

Valerio Capraro, Giorgia Cococcioni

Experimental instructions: Study 1

We report only the instruction screen, the decision screen, the comprehension questions, and the measure of experience.

Time pressure condition (instruction screen)
You have been paired with another participant. You can earn additional money depending on the decision you will make in the next screen.

You will be asked to make a choice that can affect your and the other participant's outcome. The decision problem is symmetric: also the other participant is facing the same decision problem.

After the survey is completed, you will be paid according to your and the other participant's choices.

YOU WILL HAVE ONLY 10 SECONDS TO MAKE THE CHOICE. IF YOU FAIL TO ANSWER WITHIN 10 SECONDS, YOU WILL BE AUTOMATICALLY REDIRECTED TO THE NEXT PAGE AND YOU WILL NOT EARN ANY MONEY.

This is the only interaction you have with the other participant. He or she will not have the opportunity to influence your gain in later parts of the HIT.

If you are ready, go to the next page.

Time pressure condition (decision screen, containing a visible timer counting down from 5s)
You and the other participant are both given 20c. You and the other participant can transfer, independently, money to each other. Every cent you transfer, will be doubled and earned by the other participant. Every cent you do not transfer, will be earned by you.

How much do you want to transfer? (Available options: 0c, 2c, …, 20c).

Time delay condition (decision screen)
You have been paired with another participant. You can earn additional money depending on the decision you will make in the next screen.

You will be asked to make a choice that can affect your and the other participant's outcome. The

decision problem is symmetric: also the other participant is facing the same decision problem.

After the survey is completed, you will be paid according to your and the other participant's choices.

YOU WILL HAVE TO THINK FOR AT LEAST 30 SECONDS BEFORE MAKING YOUR CHOICE. USE THIS TIME TO THINK CAREFULLY ABOUT THE DECISION PROBLEM.

This is the only interaction you have with the other participant. He or she will not have the opportunity to influence your gain in later parts of the HIT.

If you are ready, go to the next page.

Time delay condition (decision screen, containing a visible timer counting up until 30s)
You and the other participant are both given 20c. You and the other participant can transfer, independently, money to each other. Every cent you transfer, will be doubled and earned by the other participant. Every cent you do not transfer, will be earned by you.

How much do you want to transfer? (Available options: 0c, 2c, …, 20c).

Comprehension questions

1. What is the choice by YOU that maximizes YOUR outcome? (Available options: transfer 0c, transfer 2c, …, transfer 20c).
2. What is the choice by YOU that maximizes THE OTHER PARTICIPANT's outcome? (Available options: transfer 0c, transfer 2c, …, transfer 20c).
3. What is the choice by THE OTHER PARTICIPANT that maximizes YOUR outcome? (Available options: transfer 0c, transfer 2c, …, transfer 20c).
4. What is the choice by THE OTHER PARTICIPANT that maximizes THE OTHER PARTICIPANT's outcome? (Available options: transfer 0c, transfer 2c, …, transfer 20c).

Measure of level of experience
To what extent have you previously participated in other studies like to this one (i.e., exchanging money with a stranger)? (responses collected using a Likert scale from 1 = "Never" to 5 = "several times").

Experimental instructions: Study 2

Stroop task

(We report only the instructions of the Stroop task for depleted participants. Instructions of the *no-depletion* condition were identical, a part from the fact that the color in which the color word was written corresponded to the actual color word. Moreover, the instructions for the Prisoner's Dilemma, its comprehension questions, and the measure of experience were the same as in Study 1).

Please type the color in which the following words are printed (below each word there was a text box. Each word was in a different screen. People failing to give the right answer were not allowed to go to the next screen):

Red

Purple

Green

White

Pink

Black

Yellow

Blue

Brown

Violet (this was actually written in white ink, which was clearly visible since the survey was on gray ground)

Yellow

Green

Black

Blue

Red (this was actually written in white ink)

Purple

White

Violet

Brown

Pink

e-hunting task

(We report only the instructions of the *depletion* condition. Stage 1 was the same for both the *depletion* and the *no-depletion* conditions. Stage 2 in the *no-depletion* condition was identical to that in the *depletion* condition, with the only difference that the task was the same as in Stage 1.)

Stage 1

Please find all letters "e" in the text below and report their number. Note that, if you type an incorrect number the survey will not let you go to the next screen (actually, we allowed errors up to 5% of the total number of letters to be found).

"These notes deal with two particular instances of such a strategy: Sofic groups are in fact the countable discrete groups that can be approximated in a suitable sense by finite symmetric groups and groups of unitary matrices. These achievements aroused the interest of an increasing number of researchers into some fundamental questions about the nature of these approximation properties. Many of such problems are to this day still open such as, outstandingly: Is there any countable discrete group that is not sofic?"

Stage 2

Please find all letters "e" in the text below which are not one letter away from another vowel belonging to the same word So, for instance, the letter "e" in the word "finite" would not count, since it is one letter away from another vowel belonging to the same word, that is, the vowel "i". Report their number in the text box below. Note that, if you type an incorrect number the survey will not let you go to the next screen (actually, we allowed errors up to 5% of the total number of letters to be found).

"We believe that this point of view, even though rarely explicitly adopted in the literature, can contribute to a better understanding of the ideas therein, as well as provide additional tools to attack many remaining open problems. The presentation is nonetheless self-contained and accessible to any student or researcher with a graduate-level mathematical background. In particular no specific knowledge of logic or model theory is required."

give-the-wrong-answer task

(We report only the instructions for the *depletion* condition. Instructions for the no-depletion condition were exactly the same, apart from the fact that subjects were asked to give the right answer, instead of the wrong one).

For each of the following questions, please tick the WRONG answer. Answer as quickly as possible. You have only five seconds for each question.

What is the name of the President of the USA? (Available answers: Barack/Bill)

How many states are in the USA? (Available answers: 50/60)

When was the Great Depression? (Available answers: 1929/1939)

What is the typical Italian dish? (Available answers: pasta/paella)

What is the most famous monument in Paris? (Available answers: Colosseo/Tour Eiffel)

When did Armstrong land on the Moon? (Available answers: 1979/1969)

If you want to see a giraffe, you go to: (Available answers: Africa/North Pole)

How many legs does the turkey have? (Available answers: two/four)

What does a vegan not eat? (Available answers: apple/seabass)

Who is Angelina Jolie's husband? (Available answers: George Clooney/Brad Pitt)

What is the name of the actor Connery? (Available answers: Keanu/Sean)

Who played in the movie Matrix? (Available answers: Keanu Reeves/Robert de Niro)

Who was the President of the USA before Obama? (Available answers: George W. Bush/George H. W. Bush)

Who was Michael Jordan? (Available answers: a soccer player/a basketball player)

2 + 2 = (Available answers: 4/8)

What movie did Meryl Streep play in? (Available answers: the devil wears Louis Vuitton/the devil wears Prada)

What is the capital of England? (Available answers: Manchester/London)

Who founded Facebook? (Available answers: Mark Zuckerberg/Steve Jobs)

What is the name of the last Grand Slam event of the year? (Available answers: US closed/US open)

What is the name of racing cyclist Armstrong? (Available answers: Lance/Neil)

What is the largest newspaper in the US? (Available answers: The Wall Street Journal/The Denver Post)

Which one is not a borough of New York City? (Available answers: Queens/Kings)

What town is Yale University located in? (Available answers: Palo Alto/New Haven)

Where is US Bullion Depository? (Available answers: Fort Knox/Fort Alamo)

When did the Second World War end? (Available answers: 1955/1945)

What is the second most spoken language in the US? (Available answers: Spanish/Italian)

Who wrote Romeo and Juliet? (Available answers: Shakespeare/Keats)

5 x 5 = (Available answers: 35/25)

What is the name of scientist Hawking? (Available answers: Anthony/Steven)

What is the largest island in the world? (Available answers: Hawaii/Greenland)
